# Lower Limb Anthropometric Profiling in Professional Female Soccer Players: A Proof of Concept for Asymmetry Assessment Using Video Analysis

**DOI:** 10.3390/ijerph20126124

**Published:** 2023-06-14

**Authors:** Kristian J. Weaver, Nicola Relph

**Affiliations:** 1 Sports Injuries Research Group, Edge Hill University, St. Helens Road, Ormskirk, Lancashire L39 4QP, UK; 2 Faculty of Health, Social Care and Medicine, Edge Hill University, St. Helens Road, Ormskirk, Lancashire L39 4QP, UK

**Keywords:** female athlete, soccer, clinical, screening

## Abstract

The objective was to evaluate the clinical joint and limb measures in professional female soccer players. The study was a cross-sectional observational design. It was a preseason clinical setting. The inclusion criteria were outfield professional female soccer players, based in the UK, competing in the highest English league. The exclusion criteria included players who had had surgery in the last six months or had missed a single training session or match due to injury in the previous three months. In terms of the outcome measures, the dependent variables were the true limb length, ankle dorsiflexion, knee flexion and extension, hip flexion, extension, internal rotation and external rotation, and straight leg raise measured using video analysis software. Additionally, passive clinical knee and ankle stability tests were conducted. The independent variables were leg dominance and playing position (defender, midfielder, and attacker). For the results, all the ROM measurements demonstrated limb symmetry (*p* = 0.621). However, there were significant main effects of the playing position on the ankle dorsiflexion and hip internal rotation, with defenders demonstrating a significantly reduced range of motion in comparison to midfielders and attackers. A notable finding from the bilateral passive stability measures was that 38.3% of players exhibited ankle talar inversion instability when using a talar tilt. In conclusion, bilateral differences do not appear to be apparent in this population; however, positional differences may occur in the ankle and hip range of motion measures. A high proportion of this population may present with passive ankle inversion instability. Future research should consider whether this leads to a higher risk of injury in this population.

## 1. Introduction

The number of women playing professional or semi-professional football in Europe has more than doubled from the 2012/13 season from 1303 players to 2853 players in 2016/2017 [1]. The number of professional players in England in the 2016/2017 season was 215 [1]. There is evidence to support a disparity in the lower limb injury rates between male and female athletes, with females potentially being at greater risk of knee ligament injury [2,3,4] but perhaps not ankle injuries [5,6,7,8].

Despite the increase in players in the women’s professional game, there are limited normative profiling data for the professional female soccer player. Therefore, it is important to report the normative profiles of professional female football players to inform the injury risk factors, given the potential financial and personal impact of injury [9,10,11]. Despite the low-cost, ease of use, and potential links to the risk of injury associated with clinical non-performance-related measures [12,13], there is a limited number of studies assessing the presentation of bilateral or positional differences within the professional female footballer, and there are often variations in the assessment methods.

Previously, intrinsic and extrinsic factors have been described as risk factors for injury [8,14,15,16]. Anatomical and hormonal risk factors are considered to be intrinsic factors, whereas environmental and biomechanical elements are often cited to be extrinsic factors [8,14]. Anthropometric characteristics fall into the category of anatomical risk factors, which may be attributed to the exercising muscle, playing position, and training status [17,18]. Furthermore, reduced range of motion or bilateral differences have been cited as risks for injury [18,19]. In the sporting environment, anthropometric measures are often limited to height, mass, and skin fold measures in male athletes [19,20]. However, the number of participants presenting with bilateral range of motion asymmetry is not clear from the published literature [18,19,20,21]. Asymmetrical stresses on specific tissues via biomechanical loading can result in increased loads and therefore lead to overuse injury [22,23,24]. Subsequently, further scrutiny is required for the prevalence of bilateral asymmetry in the professional female soccer player [25,26]. 

Generally, midfield players have been found to be significantly faster in acceleration tests [25] and have a greater aerobic capacity [26] across sexes. Anthropometrically, defenders are taller and heavier than the other male outfield positions [27]. Comparisons between sexes and sports for individual joint ROM, particularly at the hip and ankle, have been reported in relation to injury risk [28,29]. However, information on the positional effects on clinical measurements are presently unavailable for professional female footballers.

Increased mechanical ligament instability is an additional injury risk factor, which can be a symptom of chronic instability [30,31]. Instability can also contribute to osteoarthritic changes, pain, neuromuscular alterations, and variations in talar tilt angles [30] and is therefore another potential injury risk factor in athletes. As with other clinical measures, the prevalence of clinical ankle joint instability is currently unknown in female professional football players.

## 2. Objectives

The main objective of this study was to develop the anthropometric profile of professional female soccer players, identifying the bilateral ROM and positional differences. A secondary objective was to passively assess the presence of lower limb joint stability.

## 3. Methodology

### 3.1. Design

The study was a cross-sectional observational design conducted preseason in a sports injury clinic.

### 3.2. Participants

The inclusion criteria were outfield professional female soccer players, based in the UK, competing in the highest English league. The exclusion criteria included those players who had had surgery in the last six months or had missed a single training session or match due to injury in the previous three months. Ethical procedures were followed, as well as those described in the Helsinki Declaration, and approval was received from the Departmental Research Ethics Committee (SPA-REC-2016-357).

### 3.3. Procedures

The tests were conducted as part of the preseason testing of the players, reducing the risk of seasonal match fixture fluctuations. The limb dominance was determined for each player by preferential for kicking leg. The position was self-reported as either defender, midfielder, or attacker. All testing was conducted by a practitioner with eleven years of clinical experience (KW).

The participants completed three minutes of low-intensity aerobic exercise of jogging in situ with increasing pace followed by six repetitions of dynamic movements (body weight press-ups, forward lunges, and body weight squats) with the aims of increasing muscle and tendon suppleness, stimulating blood flow to the periphery, increasing muscle temperature, and to enhance free coordinated movement [32].

The *range of motion* was measured three times, in random order (Table 1), with maximal values recorded for the *true limb length* [33], *ankle dorsiflexion* [34], *knee flexion and extension* [35,36], *hip flexion* [37], *hip extension* [37], *hip internal and external rotation* [37], *and straight leg raise (SLR)* [38]. Clear verbal instruction was given to reduce any compensatory movements. The experienced practitioner positioned the patient, obtained the image, and measured the image. The images were standardised at 1 m perpendicular to the plane of movement (Canon SX50HS, 12MP). Retrospective computer photogrammetry was applied (Kinovea; Joan, Charmont and Contrib) with computer-based markers on the identified bony landmarks. This is deemed a suitable tool for range of motion measures due to the excellent reported intra-examiner reliability ICC (_1_,_1_) = 0.99 [39].

*Joint stability* measures at the knee consisted of the posterior drawer, anterior drawer, valgus stress test at 30°, and the varus stress test at 30°. The ankle joint stability measures consisted of the talar inversion and eversion tilt and the anterior drawer. All were measured passively [40] and classified as either stable or unstable, as previously described in the literature, through the clinician’s subjective assessment [30,41]. The knee ligament tests were performed with the patient supine, lying with the knee flexed to 90° for both the anterior and posterior drawer and at 30° for the varus and valgus stress tests. The passive ankle joint stability tests were assessed with the patient seated and the foot free from the plinth, with manual fixation of the tibia [30]. All testing was performed by the same clinician to improve consistency in this subjective procedure.

### 3.4. Statistical Analyses

The data were confirmed to be parametric using normality tests (*p* > 0.05). The means and 95% confidence intervals (CIs) were presented for each measurement. The age, weight, and height differences between playing positions (defender, midfielder, and attacker) were conducted using one-way ANOVAs. A 2 (leg dominance) x 3 (playing position) MANOVA was used to report the main effects for the dependent variables, the true limb length, ankle dorsiflexion, knee flexion and extension, hip flexion and extension, hip internal and external rotation, and the SLR. The interaction between the leg dominance and playing position was also considered. Between subject effects were then compared when required. Post hoc comparisons on the significant main effects of playing position were conducted using Bonferroni testing when required. The partial eta squared ($\eta_{p}^{2}$) was added to the significant *p* values. The frequencies of the laxity measures were compared using the chi-square test and an odds ratio with 95% CI. The analysis was completed in the Statistical Package for the Social Sciences (SPSS, version 24, Chicago, IL, USA). Statistically significant differences were accepted for values *p* < 0.05.

## 4. Results

The results from thirty female professional UK-based outfield soccer players (aged 23 ± 3.12 yrs, mass 61.70 ± 5.72 kg, and height 166.8 ± 6.21 cm) were analysed. There was no significant main effect of the leg dominance (*p =* 0.621) on the dependent variables (Table 2). There were also no significant interactions between the leg dominance and position (*p* > 0.05). However, there was a significant main effect of the position (*p =* 0.030, $\eta_{p}^{2}=0.272$). Specifically, there was a between subjects’ effect for the ankle dorsiflexion (*p =* 0.025, $\eta_{p}^{2}=0.146$) and the hip internal rotation (*p =* 0.002, $\eta_{p}^{2}=0.236$) (Table 3). Post hoc analysis revealed the defenders had significantly less ankle dorsiflexion compared to the midfielders (*p =* 0.046, 95% CIs 0.03 to 4.31). Regarding the hip internal rotation, the attackers had significantly greater rotation compared to the midfielders (*p =* 0.003, 95% CIs 2.73 to 15.43) and defenders (*p =* 0.004, 95% CIs 2.70 to 16.53). All other range of motion comparisons did not reach statistical significance (*p* > 0.05). There was also no difference in age (*p =* 0.782), height (*p =* 0.649), or weight (*p =* 0.923) between playing positions.

Twelve players exhibited passive clinical talar inversion instability on their dominant side (40%), and eleven exhibited passive clinical talar inversion instability on their nondominant side (36.7%) (Table 4), with eleven players displaying bilateral instability. The odds of having an unstable ankle on the dominant side were 1.15 times higher than the nondominant side, but the 95% CI of 0.41 to 3.26 revealed this association was not significant. Therefore, the players were just as likely to have talar inversion instability on either the dominant or nondominant side.

There was no difference between the number of players with stable and with unstable ankle talar inversion results (*p =* 0.071). Importantly, only two players reported previous injury to the side that demonstrated ligament instability on passive testing. Overall, 38.3% of the female footballers produced positive results for ankle talar inversion instability.

Three players (10%) displayed ankle eversion instability for both the dominant and nondominant ankles. Four players (13.3%) had instability on the ankle anterior drawer test in their nondominant ankle, and three players (10%) had this instability in their dominant ankles. However, there were significantly more players with ankle stability than ankle instability, as would be expected (*p* = 0.001).

No players presented with unstable knee valgus or posterior drawer, and only one player demonstrated unstable knee varus in her nondominant side. One player on the nondominant side and two players on the dominant side displayed unstable knee anterior drawer.

## 5. Discussion

The objective of this study was to provide a profile of professional female soccer players. The results indicated no bilateral ROM difference but some positional differences. There was lower limb ankle joint instability present.

### 5.1. Range of Motion

Anthropometric data on professional female soccer players are rare and often limited to compositional anthropometrics such as height, mass, age, body fat, and lean body mass and do not include the joint range of motion [42]. Bilateral imbalances of the joint range of motion have been demonstrated to be of importance when assessing athletes, as restricted movement can increase the risk of injury [43]. The goal of the current study was to investigate the bilateral and positional differences in the lower limb range of motion using video analysis in professional female soccer players. However, the results of the current study demonstrated no significant bilateral differences in the joint range of motion measures, in keeping with some of the previous literature [44]. 

There was a nonsignificant difference for hip internal rotation between the dominant and nondominant sides; however, upon further analysis, the attackers displayed greater hip internal rotation in comparison to both the midfielders and defenders. It is unclear as to the reason for this, and this should be investigated further. Previous research has linked hip asymmetry to osteitis pubis, lower back pain, sacroiliac joint dysfunction, sports hernia, and increased shearing forces across the pubic symphysis [45,46,47,48,49]. Furthermore, decreased hip internal rotation in male American football players indicated a statistically significant increased odds of an ACL injury in the ipsilateral or contralateral knee (OR 0.95, *p <* 0.001) [43], although a strong link to injury is still not established. In addition, male soccer players demonstrated significantly decreased internal, external, and total hip rotation in comparison to nonathletic control groups, with negative correlations to the years of playing and the frequency of exposure, which may indicate physiological adaptations [21]. Interestingly, the hip internal rotation in professional female soccer players was lower than that of male soccer players including those who had previously had anterior cruciate ligament reconstruction, which may have implications for kinetic changes and subsequent knee injury [21,29,50]. The pattern of female soccer attacking positions demonstrating greater hip internal rotation than other positions is in keeping with the results from male soccer [29]. 

There were significant between subjects’ effects for ankle dorsiflexion, with defenders having a reduced ROM in comparison to midfielders and attackers, which may be linked with fewer changes in direction and the manipulation of the ball. Reduced ankle dorsiflexion is linked to those who have suffered ACL injury [51], changes in knee kinematics [52], patella tendinopathy [53], and ankle sprain [54,55]. Therefore, it may be important for defenders to increase ankle flexibility. These findings deviate from the findings in male soccer players, where there were no reported ankle range of motion positional differences [29]. 

The results of the current study suggest that female professional football players do not have significant bilateral differences in lower limb ROM measures. The ROM values from this study are comparable to the findings in male elite youth soccer players, which may be attributed to equal levels of exposure to the sport and training because of the rotational components of kicking [21]; however, causation has not yet been explored.

### 5.2. Ligament Stability

An additional aim of the current study was to compare the bilateral differences in joint stability. The results of the talar inversion stress test indicated that 40% of professional female soccer players exhibited inversion instability on their dominant side and 36.7% on their nondominant side, with only two players reporting previous injury to the ankle ligaments. The role of joint instability in lower limb injury is currently unclear [56,57,58]. This suggests ankle ligament function may need further consideration in those with ankle instability.

Concerningly, chronic ankle instability is considered to be a contributing factor to conditions such as osteoarthritis [59,60,61]. A high prevalence of osteoarthritis within soccer players suggests that it may not only be players with traumatic injury who are at potential risk [62]. The possibility for increased relative talocrural translation may predispose the player to increased microtrauma during the repeated loading of the joint, which initially, may have limited signs and symptoms [63,64]. The implementation of ankle specific injury prevention programmes may go some way to addressing the risks associated with the current findings [65,66,67]. The results of the current study should be considered in future work using cohort study designs to identify whether this high prevalence of ankle instability in female professional footballers is an injury risk factor.

The clinical implications may be for position specific ankle and hip injury reduction interventions to be included due to the high prevalence of ankle ligament instability and variances in the ROM in this sample. Future studies should monitor these measurements longitudinally across a season and correlate them with injury incidence. 

### 5.3. Limitations

The testing in this study was completed at one time point, preseason; therefore, it did not account for in-season fluctuations in performance. The current study design did not report the injury incidence; hence, risk factors could not be validated. However, the measurements provided here create baselines for professional female soccer players. Finally, the testing was conducted in a clinical setting, rather than laboratory-based, but we hope this increased the ecological validity.

## 6. Conclusions

There were no anthropometric asymmetries of the lower limb in this cohort of professional female soccer players, which may be a demonstration of the equal bilateral demands of the sport and a reduced risk of injury. Significant between subjects’ effects for ankle dorsiflexion and hip internal rotation were observed that indicates the need for preseason screening to obtain individual and position-specific baseline measures. In addition, a high proportion of ankle instability was reported, which may have implications for joint health and performance. Further research should add to a growing database across positions, levels, and geographical locations, as there is still an absence of evidence to link the measures obtained to injury risk.

## Figures and Tables

**Table 1:** Range of motion procedures.

Measurement	Procedure
True limb length	Measured using a tape measure from the anterior superior iliac spine to the medial malleolus whilst supine [33].
Ankle dorsiflexion	Measured using a tape measure for the knee-to-wall test, measuring the distance from the wall to the great toe without the heel raising [34].
Knee flexion and extension	Measured between the greater trochanter of the femur, lateral epicondyle of the femur (fulcrum), and the lateral malleolus of the fibula, with the patient supine [35]. During knee flexion, the participant was instructed to maintain foot contact with the plinth [36].
Hip flexion	Measured with the participant supine between the midline of the torso, greater trochanter of the femur (fulcrum), and the lateral epicondyle of the femur, with the maintenance of a flexed knee [37].
Hip extension	Measured with the participant prone between the midline of the torso, greater trochanter of the femur (fulcrum), and the lateral epicondyle of the femur [37].
Hip internal and external rotation	Measured with the participant supine and the hip in 90 degrees of flexion. The angle was calculated between a transverse line across the anterior superior iliac spine, with the fulcrum at the midpoint of the patella, and the deviation away from the neutral position of the tibia [37].
Straight leg raise	Measured with the patient lying supine and maintaining an extended knee, with the contralateral limb in contact with the plinth. The angle was measured from the midline of the torso, greater trochanter of the femur (fulcrum), and lateral malleolus of the fibula [38].

**Table 2:** Range of motion lower limb measures for the dominant and nondominant legs (mean [95% CIs]) and MANOVA results.

Test	Dominant Leg	Nondominant Leg	MANOVA Results	
			Analysis	*p* Value & $\eta_{p}^{2}$
True limb length (cm)	88.6 [87.0 to 90.2]	88.7 [87.0 to 90.3]	Main effect for leg dominance	*p* = 0.621
Ankle dorsiflexion (cm)	10.1 [9.0 to 11.1]	10.1 [9.2 to 11.2]		
Knee flexion (°)	123.0 [120.1 to 125.0]	122.7 [120.3 to 125.0]		
Knee extension (°)	−0.2 [−1.5 to 1.0]	−1 [−2.2 to 0.1]	Main effect for position	*p =* 0.030 $\eta_{p}^{2}=0.272$
Hip flexion (°)	109.8 [105.8 to 113.8]	112.8 [109.8 to 115.8]		
Hip extension (°)	20.2 [17.9 to 22.6]	20.7 [18.2 to 23.1]		
Hip internal rotation (°)	22.8 19.9 to 25.6]	25.9 [23.0 to 29.0]	Leg dominance X position interaction	*p >* 0.05
Hip external rotation (°)	20.2 [17.9 to 22.6]	20.7 [18.2 to 23.1]		
Straight leg raise (°)	87.1 [80.1 to 94.0]	87.5 [80.9 to 94.1]		

**Table 3:** Anthropometric lower limb measures by position (mean [95% CIs). * Significant main effect *p* < 0.05 level, ** significant main effect at *p* < 0.01 level. ^#^ Missing data—due to the lack of ability to accurately identify the bony landmarks from the images. NS = *p* > 0.05.

Test	Defender ^#^ (n = 16 Limbs)	Midfielder ^#^	Attacker ^#^	
		(n= 27 Limbs)	(n = 10 Limbs)	ηp2
Age (years)	22.3 [20.2 to 24.6]	23.0 [21.2 to 24.8]	22.0 [18.1 to 26.0]	NS
Height (cm)	165.1 [158.8 to 171.4]	167.8 [164.1 to 171.5]	165.7 [158.9 to 172.5]	NS
Weight (kg)	61.1 [55.9 to 66.4]	60.7 [57.9 to 63.5]	61.9 [55.1 to 68.8]	NS
Ankle dorsiflexion (cm) *	8.5 [7.0 to 10.1]	10.7 [9.7 to 11.7]	11.2 [9.3 to 13.0]	0.146
Knee flexion (°)	123.6 [119.2 to 128.0]	121.5 [118.6 to 124.4]	123.5 [118.9 to 128.1]	NS
Knee extension (°)	−0.1 [−2.0 to 1.8]	−1.2 [−2.4 to 0.05]	−0.7 [−2.5 to 1.1]	NS
Hip flexion (°)	110.2 [105.4 to 115.0]	112.4 [109.5 to 115.3]	112.5 [104.4 to 120.6]	NS
Hip extension (°)	20.5 [16.4 to 24.6]	19.9 [17.2 to 22.7]	21.8 [18.0 to 25.6]	NS
Hip internal rotation (°) **	22.7 [19.0 to 16.4]	23.2 [20.3 to 26.2]	32.3 [27.9 to 36.7]	0.236
Hip external rotation (°)	29.7 [24.0 to 35.4]	28.4 [24.4 to 32.4]	34.5 [29.5 to 39.5]	NS
Straight leg raise (°)	91.1 [79.3 to 102.9]	86.7 [79.5 to 93.8]	79.4 [67.9 to 90.9]	NS

**Table 4:** The relationship between the clinical talar inversion stability and the limb dominance.

		Limb Dominance		
		Dominant Side	Nondominant Side	Total
Talar Inversion	Unstable	12	11	23
	Stable	18	19	37
Total		30	30	60

## Data Availability

Data supporting reported results is available on request from the corresponding author.
